# Effectiveness of Amiodarone in Preventing the Occurrence of Reperfusion Ventricular Fibrillation After the Release of Aortic Cross-Clamp in Open-Heart Surgery Patients: A Meta-Analysis

**DOI:** 10.3389/fcvm.2022.821938

**Published:** 2022-02-04

**Authors:** Li-min He, An Zhang, Bin Xiong

**Affiliations:** ^1^Department of Cardiology, The First Affiliated Hospital, Chongqing Medical University, Chongqing, China; ^2^Department of Critical Care Medicine, The Second Affiliated Hospital, Chongqing Medical University, Chongqing, China

**Keywords:** amiodarone, reperfusion ventricular fibrillation, aortic cross clamp, open-heart surgery, meta-analysis

## Abstract

**Objective:**

To evaluate the efficiency of amiodarone in preventing the occurrence of reperfusion ventricular fibrillation (RVF) after aortic cross-clamp (ACC) release in patients undergoing open-heart surgery.

**Methods:**

We searched the Web of Science, Cochrane Library, EMBASE, and PubMed databases through January 2021 for relevant studies addressing the efficacy of amiodarone in preventing RVF after ACC release in patients undergoing cardiac surgery. A complete statistical analysis was performed using RevMan 5.3. Risk ratios (RRs) and 95% confidence intervals (CIs) were calculated to express the results of dichotomous outcomes using random or fixed-effect models. The chi-square test and *I*^2^ test were used to calculate heterogeneity.

**Results:**

Seven studies (856 enrolled patients; 311 in the amiodarone group, 268 in the lidocaine group, and 277 in the placebo group) were selected for the meta-analysis. The incidence of RVF was significantly decreased in the amiodarone group compared to the placebo group (RR = 0.69, 95%CI: 0.50–0.94, *P* = 0.02). However, amiodarone and lidocaine did not confer any significant difference (RR = 0.98, 95%CI: 0.61–1.59, *P* = 0.94). The percentage of patients requiring electric defibrillation counter shocks (DCSs) did not confer any significant difference between patients administered amiodarone and lidocaine or placebo (RR = 1.58, 95%CI: 0.29–8.74, *P* = 0.60; RR = 0.55, 95%CI: 0.27–1.10, *P* = 0.09; respectively).

**Conclusions:**

Amiodarone is more effective than a placebo in preventing RVF after ACC release in cardiac surgery. However, the amiodarone group required the same number of electrical DCSs to terminate RVF as the lidocaine or placebo groups.

## Introduction

Open-heart surgery is a primary treatment option for patients with severe valvular heart disease (VHD) and multiple-vessel coronary heart disease (CHD) (1–3). However, patients undergoing valve replacement surgery or coronary artery bypass surgery (CABG) are prone to risks associated with surgical complications such as arrhythmias, major bleeding, severe infection, and cerebral infarction (4). Ventricular fibrillation (VF) frequently occurs after aortic cross-clamp (ACC) release in patients undergoing open-heart surgery (5–7), which can result in reperfusion ventricular fibrillation (RVF) when myocardium reperfusion is initiated. This surgical consequence is associated with a negative impact on morbidity and mortality (5–7).

The etiopathogenesis of RVF is explained through several mechanisms that occur in combination with myocardial ischemia-reperfusion injury (IRI), including subsequent auto-inflammatory responses, oxidative stress, and electrical instability, which may lead to sudden cardiac death. Oxidative stress influences ion homeostasis due to changes in the ion channel structure and function, which play a critical role in increased levels of circulating catecholamine and angiotensin II that consequently elicit ventricular arrhythmias, such as VF (8). Previous studies have demonstrated that RVF is most frequently associated with post ACC release during the CABG procedure. Earlier studies have reported that the incidence of RVF occurrence after ACC release ranges between 45 and 100% (9–11), but a more recent study reported that the rate is between 10 and 80% (7). This variation in RVF incidence indicates a change in the type of cardiac operation and the experience and skills of the surgeon (6).

External electric defibrillation is sometimes used to terminate RVF and achieve normal cardiac impulse transmission through the heart's electrical conduction system. When defibrillation is delayed, effectiveness is reduced by almost 10% per minute. However, attempting to provide electric shock may result in coronary ischemia or acute myocardial infarction, further worsening the patient's condition (12). To overcome this, it has been recommended that patients undergoing cardiac surgery be administered anti-arrhythmic drugs, such as amiodarone or lidocaine, during the perioperative period to effectively prevent RVF after ACC release (6, 13).

A comparative study of amiodarone administration with placebo before ACC release was shown to significantly reduce RVF occurrence in patients undergoing cardiac surgery (5, 9), but other studies have reported contradictory results (10, 11, 14, 15). Comparisons between amiodarone and lidocaine have also shown similar contradictory results (5, 7, 10, 11, 15). This study aimed to explore the effectiveness of amiodarone in preventing RVF after ACC release in open-heart surgery patients.

## Materials and Methods

This meta-analysis was conducted and reported according to the instructions and recommendations provided in the Preferred Reporting Items for Systematic Reviews and Meta-Analyses (PRISMA) 2020 guidelines (16).

### Inclusion and Exclusion Criteria

A comprehensive search of the literature was performed for this systematic review. Our search criteria included: (1) randomized controlled trials (RCTs), (2) all enrolled adult patients who required ACC after undergoing open-heart surgery, (3) patients were randomly divided into the placebo group, lidocaine group, or amiodarone group, and (4) primary outcome measurements were included with the incidence of RVF, and percentage of patients requiring electric defibrillation counter shocks (DCSs).

Exclusion criteria were as follows: (1) non-open-heart surgery, (2) animal research, (3) comments, correspondences, case reports, and reviews, and (4) detailed outcome results of the previous studies, which were not reported.

### Information Source and Search Strategy

We initially searched for all relevant studies, without any language limitations, through January 2021. We extensively searched the published literature using the following databases: The Cochrane Library using the Cochrane Handbook for Systematic Reviews of Interventions 5.0.2, Web of Science, EMBASE, and PubMed. The PubMed, Ovid, and Cochrane databases were used to identify peer-reviewed original research articles by applying the keywords and MESH headings as follows: “amiodarone” AND “cordarone,” “ventricular fibrillation” OR “ventricular arrhythmia,” “reperfusion ventricular fibrillation” AND “reperfusion ventricular arrhythmia,” “open heart surgery” OR “cardiac surgery.” A search was run as “cited reference studies” using the Cochrane Collaboration database to cite the appropriate studies and authors as references to check for the study's relevance. A manual search of the bibliography was also performed.

### Data Extraction and Quality Evaluation

The searched research articles were thoroughly screened again and rechecked by two independent reviewers, He and Xiong, who were paired based on their educational background to ensure that at least one reviewer had clinical expertise and one reviewer had research experience. Both reviewers were informed of the inclusion and exclusion criteria to ensure proper selection of screened titles and abstracts of relevant articles from the database searches. The reviewers independently obtained the required data regarding patient demographics, left ventricular ejection fraction (LVEF), type of surgery performed, duration of ACC, cardiopulmonary bypass (CPB), and some relevant parameters during CPB (Table 1), and they evaluated whether the quality of the content in the article matched the title selected.

Table 1Demographic characteristics and intraoperative data of the included studies.

**Table:** 

**Study**	**Area**	**Study design**	**Subjects**	**Male**	**Age**	**LVEF (%)**	**Operation type**
Ayoub (11)	America	RCT	A: 40 L: 40 P: 40	A: 36 L: 37 P: 36	A: 63 ± 9 L: 64 ± 9 P: 65 ± 10	A: > 35 L: > 35 P: > 35	CABG
Samantaray et al. (9)	India	RCT	A: 17 P: 17	A: 11 P: 12	A: 47.2 ± 6.6 P: 50.0 ± 6.0	A: > 35 P: > 35	CABG
Kar (14)	India	RCT	A: 28 P: 28	A: 16 P: 14	A: 36.89 ± 12.14 P: 35.25 ± 8.4	NA	Valve
Mauermann et al. (10)	America	RCT	A: 115 L: 115 P: 112	A: 74 L: 81 P: 82	A: 63.3 ± 13.6 L: 62.7 ± 13.9 P: 63.6 ± 13.0	A: 62.7 ± 10.8 L: 62.7 ± 11.5 P: 62.8 ± 11.7	CABG, Valve, and Myectomy
Alizadeh-Ghavidel (15)	Iran	RCT	A: 50 L: 50 P: 50	A: 39 L: 40 P: 43	A: 58.06 ± 10.47 L: 60.64 ± 15.62 P: 57.43 ± 10.97	A: 42.65 ± 6.80 L: 43.64 ± 6.93 P: 43.52 ± 7.36	CABG
Yilmaz et al. (5)	Turkey	RCT	A: 27 L: 29 P: 30	A: 22 L: 22 P: 24	A: 57.2 ± 7.9 L: 61.6 ± 8.6 P: 59.7 ± 9.8	A: 53.2 ± 10.3 L: 52.8 ± 9.0 P: 52.5 ± 9.0	CABG
Mita et al. (7)	Japan	RCT	A: 34 L: 34	A: 21 L: 19	A: 70.5 ± 7.8 L: 71.5 ± 8.8	A: 65.5 ± 7.9 L: 62.4 ± 11.8	Valve

**Table:** 

**Study**	**pH**	**ACC time (min)**	**CPB time (min)**	**Core temperature (time of cross clamp release) (** **°** **C)**
Ayoub (11)	A: 7.40 ± 0.05 L: 7.41 ± 0.03 P: 7.41 ± 0.04	A: 42.0 ± 20.0 L: 44.0 ± 22.0 P: 35.0 ± 11.0	A: 66.0 ± 32.0 L: 67.0 ± 33.0 P: 64.0 ± 29.0	29
Samantaray et al. (9)	NA	A: 49.4 ± 12.3 P: 48.8 ± 15.2	A: 76.1 ± 16.5 P: 74.2 ± 18.7	30
Kar (14)	NA	A: 63.78 ± 8.6 P: 63.78 ± 10.5	A: 101.25 ± 12.3 P: 108.89 ± 11.4	NA
Mauermann et al. (10)	NA	A: 47.4 ± 32.1 L: 46.5 ± 56.1 P: 53.3 ± 36.8	A: 70.8 ± 64.0 L: 74.3 ± 40.0 P: 78.0 ± 48.7	32
Alizadeh-Ghavidel (15)	A: 7.35 ± 0.07 L: 7.34 ± 0.06 P: 7.36 ± 0.06	A: 38.2 ± 19.6 L: 35.6 ± 12.6 P: 34.9 ± 14.0	A: 72.8 ± 29.2 L: 72.1 ± 21.2 P: 65.1 ± 29.7	34
Yilmaz et al. (5)	A: 7.40 ± 0.04 L: 7.50 ± 0.51 P: 7.40 ± 0.06	A: 67.6 ± 19.7 L: 64.1 ± 18.9 P: 63.2 ± 8.8	A: 104.1 ± 31.3 L: 113.6 ± 27.8 P: 114.4 ± 27.6	34
Mita et al. (7)	A: 7.36 ± 0.04 L: 7.36 ± 0.04	A: 135 ± 44 L: 148 ± 42	A: 165 ± 51 L: 188 ± 52	36

**Table:** 

**Study**	**Timing of electrical defibrillation**	**Timing of the medication given before ACC release (min)**	**Dose of the medication given**
Ayoub (11)	DCSs immediately after RVF	A: 2 L: 2 P: 2	A: 150 mg L: 100 mg P: constant volume
Samantaray et al. (9)	DCSs immediately after RVF	A: 3 P: 3	A: 150 mg P: constant volume
Kar (14)	NA	NA	A: 3mg/kg P: constant volume
Mauermann et al. (10)	DCSs immediately after RVF	A: 3 L: 3 P: 3	A:300 mg: L: 1.5 mg/kg P: constant volume
Alizadeh-Ghavidel (15)	DCSs immediately after RVF	A: 3 L: 3 P: 3	A: 150 mg L: 100 mg P: constant volume
Yilmaz et al. (5)	RVF persist untreated for 2 min	A: 15 L: 2 P: NA	A: 300 mg L: 1.5 mg/kg P: constant volume
Mita et al. (7)	DCSs immediately after RVF	NA	A: 150 mg L: 1mg/kg

**Table:** 

**Study**	**Potassium (mEq/L) (during cross clamp)**	**Cardioplegia solution**
Ayoub (11)	A: 4.7 ± 1.0 L: 4.8 ± 0.5 P: 4.9 ± 0.5	Crystalloid hyperkalemic cardioplegia
Samantaray et al. (9)	A: 4.4 ± 0.3 P: 4.5 ± 0.5	Crystalloid hyperkalemic cardioplegia
Kar (14)	NA	St. Thomas' solution-based crystalloid-blood cardioplegic solution
Mauermann et al. (10)	NA	Crystalloid hyperkalemic cardioplegia
Alizadeh-Ghavidel (15)	A: 4.10 ± 0.44 L: 4.11 ± 0.42 P: 4.24 ± 0.47	Retrograde St. Thomas solution
Yilmaz et al. (5)	A: 5.00 ± 0.63 L: 4.80 ± 0.61 P: 4.40 ± 0.89	Retrograde St. Thomas solution and crystalloid hyperkalemic cardioplegia
Mita et al. (7)	A: 4.9 ± 0.4 L: 5.0 ± 0.5	Crystalloid hyperkalemic cardioplegia

*A, amiodarone group; L, lidocaine group; P, placebo group*.

*ACC, aortic cross-clamp; CABG, coronary artery bypass graft; CPB, cardiopulmonary bypass; DCSs, defibrillation counter shocks; LVEF, left ventricular ejection fraction; Myectomy, septal myectomy; NA, not available; RCT, randomized controlled trial; RVF, reperfusion ventricular fibrillation; Valve, valve surgery*.

*Data are presented as mean ± standard deviation (SD)*.

The full texts of the potentially relevant studies were retrieved for a full review. The reviewers further discussed and resolved any discrepancies regarding the eligibility of studies; unresolved discrepancies were brought to the third author (Zhang) for a decision. Additional articles were browsed based on internal references, and appropriate information was obtained. The reviewers crosschecked all articles and made a final list of references, and discussed the list with the third reviewer before writing this systematic review.

We used the Cochrane Back Review Group 12-item scale to evaluate the quality of all included studies (17). The 12 items included: adequate sequence generation; concealment of allocation; blinding (patient); blinding (investigator); blinding (assessor); incomplete outcome data addressed (dropouts); incomplete outcome data addressed (intention-to-treat (ITT) analysis); free of selective reporting; similarity at baseline; co-interventions constant; compliance acceptable; and similar timing outcome assessments (6, 18). Studies with more than seven items were scored as “High”; those with 4 to 7 items were scored as “Moderate”; those with no more than 4 items were scored as “Low” (Table 2).

**Table 2:** Methodological quality of the included studies based on the 12-item scoring system.

**Study**	**A^**1**^**	**B**	**C**	**D**	**E**	**F^**2**^**	**G^**3**^**	**H**	**I**	**J**	**K**	**L**	**Quality^**4**^**
Ayoub (11)	–	–	+	?	?	+	+	+	+	+	+	+	High
Samantaray et al. (9)	–	+	+	?	?	+	+	+	+	+	+	+	High
Kar (14)	–	–	+	?	?	+	+	+	+	+	+	+	High
Mauermann et al. (10)	–	–	+	?	?	+	+	+	+	+	+	+	High
Alizadeh-Ghavidel (15)	–	–	+	?	?	+	+	+	+	+	+	+	High
Yilmaz et al. (5)	–	–	+	?	?	+	+	+	+	+	+	–	Moderate
Mita et al. (7)	–	–	+	?	?	+	+	+	+	+	+	–	Moderate

*(A), adequate sequence generation; (B), concealment of allocation; (C), blinding (patient); (D), blinding (investigator); (E), blinding (assessor); (F), incomplete outcome data addressed (dropouts); (G), incomplete outcome data addressed (ITT analysis); (H), free of selective reporting; (I), similarity at baseline; (J), cointerventions constant; (K), compliance acceptable; (L), similar timing outcome assessments. +: yes; –: no; ?: unclear*.

1 *Only if the sequencing method was explicitly introduced was a study given a “+”; sequence generated by “Dates of Admission” or “Patient's Number” received a “–”*.

2 *Drop-out rates <20% were given a “+”, otherwise “–”*.

3 *ITT, intention-to-treat; only if all randomized participants were analyzed in the group to which they were allocated was a study given a “+”*.

4 *Studies with more than 7 items were scored as “High”; those with more than 4, but no more than 7 items were scored as “Moderate”; those with no more than 4 items were scored as “Low”*.

### Statistical Analysis

RevMan 5.3 was used for statistical analysis. The chi-square test and *I*^2^ test were performed to calculate the heterogeneity of the sample size. Heterogeneity was considered high, moderate, or low for the estimated *I*^2^ values of 75, 50, and 25%, respectively. If low heterogeneity was found, the fixed effect method was performed; if not, the random effect model was performed. Subgroup analyses were used to evaluate the potential source of heterogeneity. Publication bias was assessed using a funnel plot. The statistical significance was considered if the *P*-value was < 0.05 (*P* < 0.05).

## Results

### Study Characteristics

**Figure 1:**
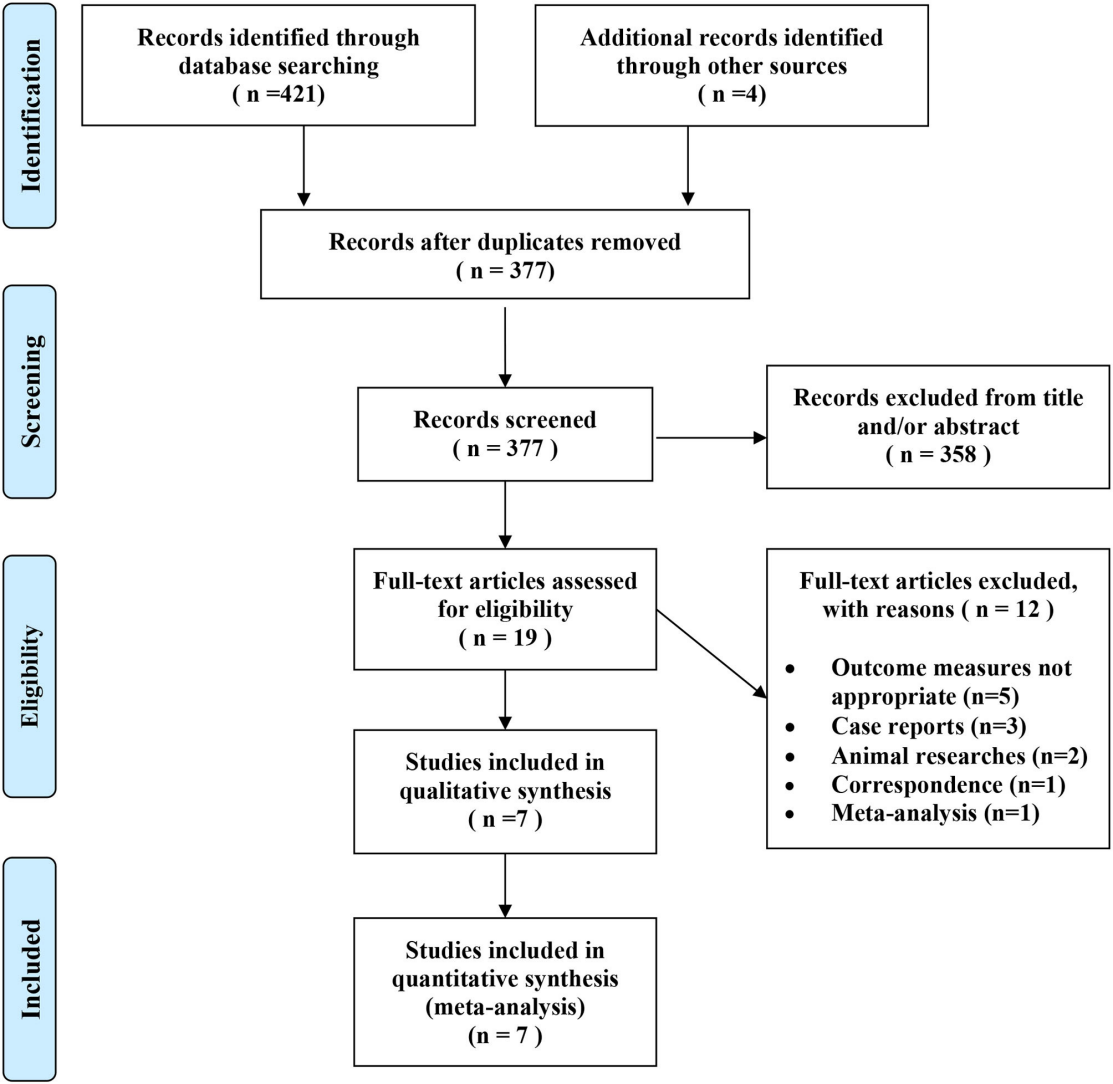
Selection of randomized controlled trials for this meta-analysis.

We identified 425 potentially relevant articles from the electronic databases as described earlier. According to the inclusion criteria, 19 articles were retrieved and needed further evaluation after screening the title or abstract. We conducted a full-text review of the previous articles, and 12 articles were excluded for not meeting the inclusion criteria. Finally, 7 RCTs (5, 7, 9–11, 14, 15) were included in this analysis. Figure 1 shows the selection process.

A total of 856 participants were enrolled in the 7 RCTs (5, 7, 9–11, 14, 15); 311 patients were included in the amiodarone group, 268 in the lidocaine group and 277 in the placebo group. According to the quality evaluation standard, 5 of the included studies met high-quality criteria, and the remaining 2 were medium quality (Table 2). Among these studies, 1 study compared the efficiency of both amiodarone and lidocaine (7), 2 studies compared the effectiveness of amiodarone with placebo (9, 14), and 4 studies were based on three-arm trials (5, 10, 11, 15) comparing amiodarone vs. placebo vs. lidocaine. All patients were matched for gender and age, underwent elective cardiac surgery, had LVEF, and matched the operative condition. In 4 studies, patients underwent CABG (5, 9, 11, 15), 2 had patients who had valve surgery (7, 14), and the other included patients who underwent CABG, valve surgery, and septal myectomy (10) (Table 1).

### Quantitative Data Synthesis

**Figure 2:**
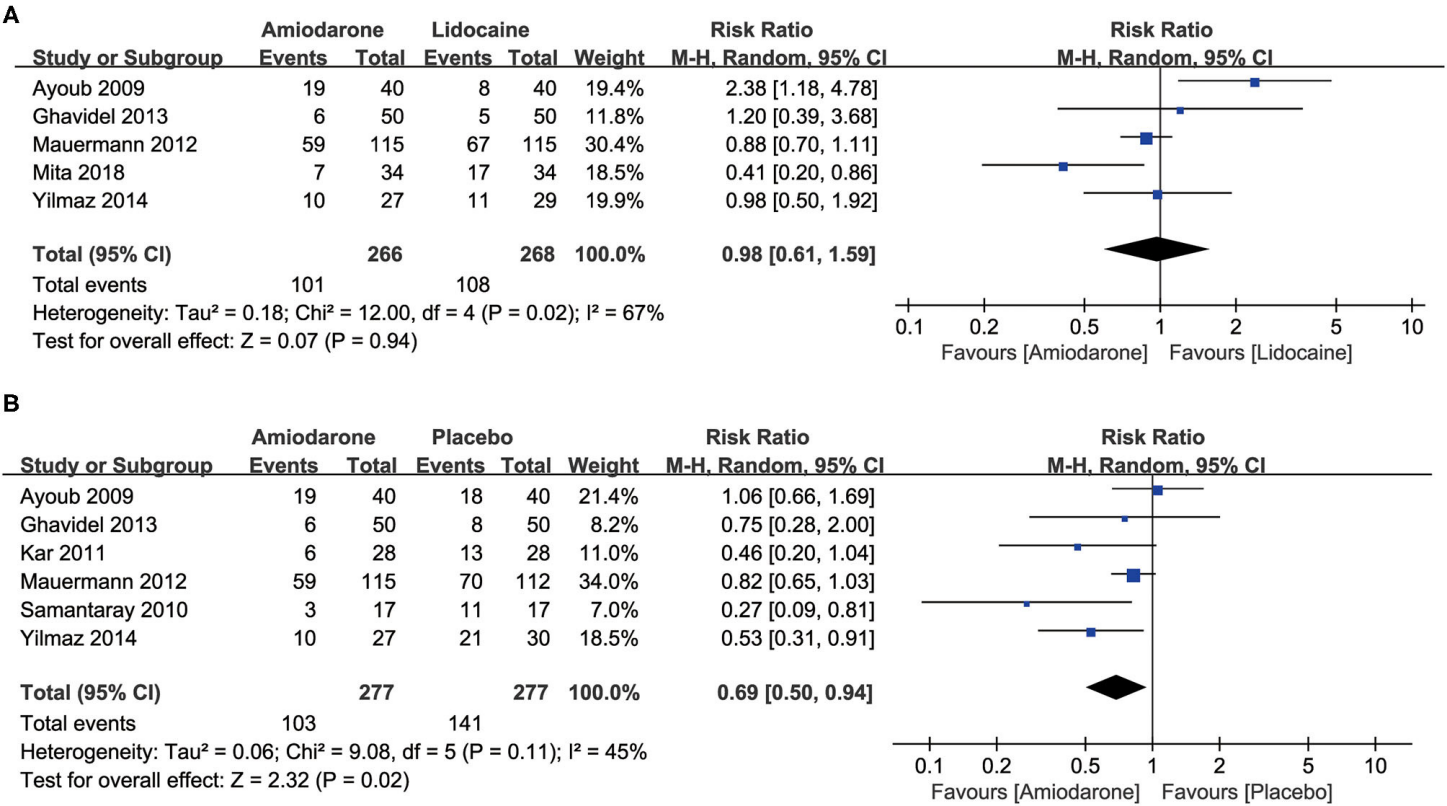
Forest plot comparing the incidence of ventricular fibrillation (VF) after the release of aortic cross-clamp (ACC) in patients undergoing open-heart surgery who were treated with amiodarone or lidocaine or placebo. CI, confidence intervals. The rate of VF after release of ACC did not differ significantly between patients undergoing open heart surgery who were treated with amiodarone or lidocaine **(A)**; amiodarone was associated with a lower risk of VF than placebo **(B)**.

Five studies (5, 7, 10, 11, 15) compared the efficacy of amiodarone and lidocaine on the incidence of RVF after ACC release in patients undergoing cardiac surgery. We found that the RVF occurrence rate after ACC release was significantly decreased in the amiodarone group compared to the placebo group (risk ratio (RR) = 0.69, 95% confidence interval (CI): 0.50–0.94, *P* = 0.02; Figure 2B) (5, 9–11, 14, 15). However, there was no significant difference between patients administrated amiodarone and lidocaine (RR = 0.98, 95%CI: 0.61–1.59, *P* = 0.94; Figure 2A).

**Figure 3:**
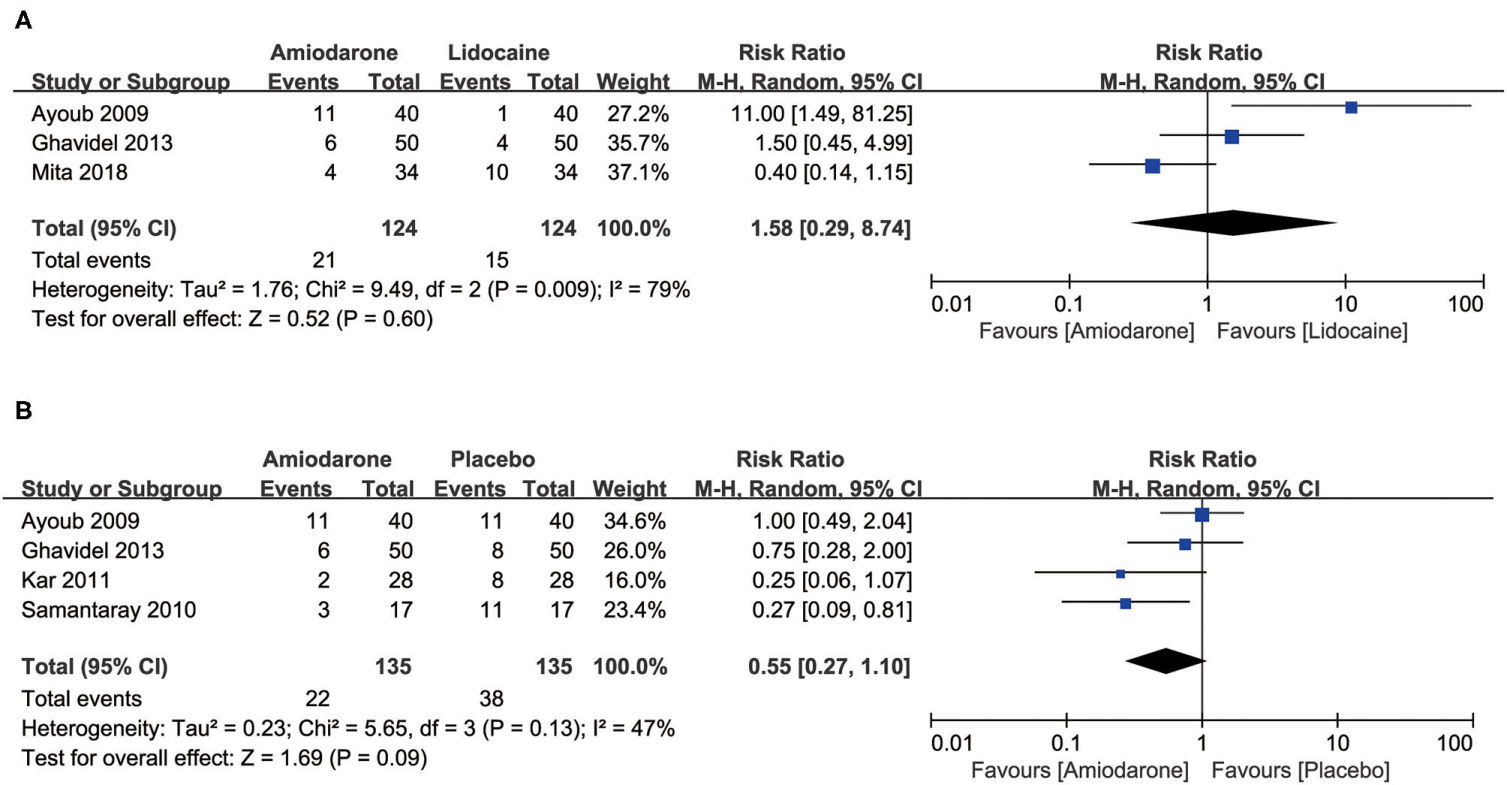
Forest plot comparing the ratio of patients who subsequently required defibrillation counter shocks (DCSs) after the release of aortic cross-clamp (ACC) during open-heart surgery. CI, confidence intervals. The rate did not differ significantly between amiodarone and lidocaine groups **(A)**; the percentage of patients requiring DCSs for VF did not differ significantly between patients receiving amiodarone and placebo **(B)**.

Four studies compared the placebo group (270 patients) (9, 11, 14, 15) and found that the percentage of patients requiring DCSs for RVF was decreased but did not confer any statistical significance in the amiodarone group with moderate heterogeneity (RR = 0.55, 95%CI: 0.27–1.10, *P* = 0.09; Figure 3B). However, 3 studies (7, 11, 15) that included 248 patients found no significant difference in the rate of patients requiring DCSs for RVF between the amiodarone and lidocaine groups (RR = 1.58, 95%CI: 0.29–8.74, *P* = 0.60; Figure 3A).

### Heterogeneity Analysis

The existence of heterogeneity among trials was evaluated in this meta-analysis. The heterogeneity test was conducted for those studies in which an individual study was excluded. Moreover, due to the scaling between moderate to high heterogeneity in all total pooled consequences, the random effect model was performed to combine the effect size.

Sensitivity analysis was subsequently performed, which was based on the type of surgery. Compared to the lidocaine group, we found the occurrence rate of RVF was higher in 3 studies (5, 11, 15) consisting of 236 patients (RR = 1.44, 95%CI: 0.79–2.62, *P* = 0.23; Supplementary Figure 1A). The percentage of patients requiring DCSs for RVF was observed in 2 studies (11, 15) consisting of 180 patients (RR = 3.49; 95%CI: 0.46–26.65, *P* = 0.23; Supplementary Figure 2A), with no significant difference in the amiodarone group. We found a similar outcome between the amiodarone group and placebo group in 4 studies (5, 9, 11, 15) consisting of 271 patients (RR = 0.64; 95%CI: 0.38–1.10, *P* = 0.11), and in 3 studies (9, 11, 15) consisting of 214 patients (RR = 0.64; 95%CI: 0.31–1.34, *P* = 0.24), respectively (Supplementary Figures 1B, 2B).

Due to the small number of patients included in these studies, meta-regression analyses were not performed, and evaluation of the publication bias was difficult to estimate.

## Discussion

Reperfusion-induced VF after removal of ACC is a significant complication of open-heart surgery. We conducted a meta-analysis to determine the efficiency of amiodarone compared to placebo or lidocaine for significantly reducing the incidence of RVF after ACC release during cardiac surgery. Our analysis showed that amiodarone is more effective than placebo for preventing RVF occurrence, while amiodarone and lidocaine confer comparable preventative efficacy. However, the efficacy of amiodarone was comparable to lidocaine and placebo for preventing RVF in patients subsequently requiring DCSs.

Our analysis showed that RVF incidence after ACC release during open-heart surgery was comparatively high, related to poor prognosis. Recent studies have shown that anti-arrhythmic drugs can effectively prevent RVF during open-heart surgery (11, 19). Lidocaine and amiodarone are commonly anti-arrhythmic clinical medicines (20, 21) because they exert sympatholytic, sodium, and calcium antagonistic properties that decrease conduction through the atrioventricular (AV) node and sinus node. Currently, only a few studies have directly compared the effects of these two drugs on RVF after ACC release during open-heart surgery. A study by Rea et al. (22) compared the combination of amiodarone and lidocaine in 194 patients suffering from in-hospital sudden cardiac arrest (IHCA). A Cox regression analysis of patients given amiodarone showed a lower probability of survival after 24 h and survival to hospital discharge than patients treated with lidocaine. However, these findings cannot determine if the poor outcome of the treatment is due to amiodarone treatment or due to a longer duration of cardiopulmonary resuscitation (CPR). Amiodarone treatment could be time-sensitive, suggesting that the earlier amiodarone is administered during CPR, the more effective the outcomes. Therefore, amiodarone is more effective than lidocaine when given early during CPR (23).

Our meta-analysis shows that lidocaine and amiodarone have similar efficacy in preventing VF occurrence after ACC release during cardiac surgery, consistent with previous studies (6, 10, 15). A previous study showed that a constant supply of a magnesium lidocaine mixture into the bloodstream of patients prevents the re-onset of impulsive electrical activity. This study also showed that conduction frequency and post-CPB ventricular arrhythmias occurred in spontaneous VF after ACC release compared to the control group undergoing CABG surgery. Administration of lidocaine alone as an intravenous injection bolus or in combination infusion before ACC release has shown contradictory results in various RCT studies. Based on trial reports, lidocaine can effectively reduce the occurrence of reperfusion VF to 84%, but in others, no reduction was observed (24). Another study also suggested that lidocaine may be better than amiodarone (11), in that amiodarone can stabilize myocardial cells and has been widely used to prevent and treat arrhythmias (25). An experimental study by Zoerner et al. provides insight into the potential mechanism of amiodarone in preventing VF during open-heart surgery. They found that in a pig model of bleeding-induced VF, combined resuscitation with vasopressin and amiodarone after hemorrhagic circulatory arrest resulted in greater 3-h survival, better preserved hemodynamic parameters, and smaller myocardial injury compared to resuscitation with vasopressin only, indicating that amiodarone terminated VF (26). In addition, early defibrillation is advisable according to the American Heart Association (AHA) guidelines. Moreover, prolonged hypoperfusion may result in intramyocardial acidosis and end-organ damage over a period of time. Early amiodarone administration is recommended to prevent such damage to maintain the spontaneous perfusing rhythm, terminate VF, and improve neurological outcomes at hospital discharge. This recommendation is in accordance with the 2018 AHA guidelines, which state that amiodarone is beneficial at the early onset of disease. (27). The results of this analysis are also by previous research that indicated that amiodarone might be related to a lower long-term ventricular defibrillation threshold (6, 28, 29), suggesting that amiodarone has beneficial effects on malignant arrhythmia. Therefore, the usage of amiodarone to treatment arrhythmias has been affirmed.

Our meta-analysis shows that amiodarone is more effective than a placebo for preventing RVF occurrence. As we know, an increase in coronary blood supply after ACC release may aggravate myocardial damage, causing IRI, which can manifest as severe or even fatal arrhythmias such as VF, heart failure, and cardiogenic shock (30). Studies have shown that calcium ions overload the production of large amounts of oxygen free radicals, and the secretion of endothelial factors from vascular endothelial cells may be related to this pathophysiological process (31). Activation of neutrophils, increased myocardial autonomy, an elevated VF threshold in the ischemic myocardium, and a myocardial electrolyte disturbance may also be involved in IRI occurrence. Interleukin-6 (IL-6) is a cytokine that mediates the inflammatory response and inhibits the release of inflammatory factors that can reduce the accumulation of neutrophils in microvessels, thereby reducing myocardial damage (32). Studies have shown that pro-inflammatory cytokine IL-6 levels are higher in patients after cardiopulmonary bypass, which may be one causal factor of IRI in these patients. This mechanism has been demonstrated by blocking mitochondrial DNA accumulation in the circulation in various *in vivo* bypass models, such as post-sternotomy/cardiopulmonary. Another study reported that blocking Toll-like receptor 9 (TLR-9) subsequently resulted in low IL-6 production in an *in vivo* model. This evidence confirms a direct relationship between TLR-9 signaling and subsequent IL-6 driven inflammatory pathways in surgical trauma. Hence, reducing IL-6 levels can be an effective treatment strategy for cardiopulmonary bypass patients (33). Studies have shown that amiodarone can improve the level of inflammatory factors by increasing the activity of ion channels and inhibiting the Na^+^/Ca^2+^ exchange protein, thereby reducing calcium overload during blood reperfusion without aggravating deterioration of heart function and by preventing reperfusion arrhythmia (34).

In this study, we found no significant difference in the proportion of patients who subsequently required DCS to terminate VF following open-heart surgery between amiodarone and lidocaine or placebo treatment groups. This result is inconsistent with the reported incidence of VF after cardiac surgery in studies that investigated DCS. However, the statistical power of the pooled analysis was limited.

## Limitations

Our study has the following limitations. Firstly, we included only the results of 7 RCTs that enrolled 856 patients undergoing cardiac surgery. The sample size of each included trial was small, which restricted further analyses of other parameters that may influence outcomes, such as study area, comorbidities, ACC time, CPB time, etc. Therefore, more large-scale RCTs are needed to verify our results. Secondly, whether the random sequence generation and outcome measurement of the included studies are blinded is uncertain, resulting in moderate quality of some studies. Thirdly, there was significant heterogeneity in our study. Sensitivity analyses were performed, but heterogeneity was significant despite excluding individual studies. Fourthly, the optimal regimen and amiodarone dosages in the perioperative period were not uniform among the included studies. Fifthly, we had only research articles in English, leading to the potential of bias. Finally, there is no recommended time for amiodarone before releasing ACC to prevent RVF, and the enrolled studies have it at different times. Five studies (5, 9–11, 19) reported the timing of amiodarone administration before ACC release. Three studies (9, 10, 19) reported amiodarone administration 3 minutes before the removal of the ACC, one study (11) reported administration 2 min before ACC release, and another study (10) reported administration 15 min before ACC release.

## Clinical and Research Implications

These results of our meta-analysis may highlight the potential use of conventional antiarrhythmic medications to prevent RVF during cardiac surgery procedures. Further studies with more significant numbers of participants are needed to confirm our results and evaluate the time-sensitivity of amiodarone in preventing RVF post ACC removal and determine the standardized protocol and regimens for the perioperative administrations of amiodarone during open-heart surgery.

## Conclusions

Amiodarone is more effective than a placebo in preventing RVF after ACC release in cardiac surgery. However, the amiodarone group required the same number of electrical DCSs to terminate RVF as the lidocaine or placebo groups. In addition, we would also highlight that it indicates the need for further studies to establish if the use of amiodarone is time-sensitive and whether this may further reduce the risk of RVF with consequent DCSs.

## Data Availability Statement

The original contributions presented in the study are included in the article/Supplementary Material, further inquiries can be directed to the corresponding author/s.

## Author Contributions

L-mH carried out the analysis and interpretation of data and participated in drafting and editing the manuscript. BX submitted the manuscript. The articles were reviewed by L-mH and BX independently as per the inclusion criteria. The quality of the articles was assessed by L-mH and BX independently, and disagreements were resolved by discussing with AZ. BX was responsible for the conception, design, and coordination of the study and revising the manuscript for important intellectual content. AZ revised the manuscript. All authors read and approved the final manuscript.

## Funding

This work was supported by the Technology Innovation and Application Development Project of Chongqing Science and Technology Bureau and Chongqing Health Commission (No. 2020FYYX212).

## Conflict of Interest

The authors declare that the research was conducted in the absence of any commercial or financial relationships that could be construed as a potential conflict of interest.

## Publisher's Note

All claims expressed in this article are solely those of the authors and do not necessarily represent those of their affiliated organizations, or those of the publisher, the editors and the reviewers. Any product that may be evaluated in this article, or claim that may be made by its manufacturer, is not guaranteed or endorsed by the publisher.

## Supplementary Material

Supplementary Figure 1 Forest plot comparing the incidence of ventricular fibrillation (VF) after the release of aortic cross-clamp (ACC) in patients undergoing coronary artery bypass surgery (CABG) who were treated with amiodarone, lidocaine or placebo. CI, confidence intervals. The rate of VF after release of ACC did not differ significantly between patients undergoing open heart surgery who were treated with amiodarone or lidocaine **(A)**; amiodarone was not associated with a lower risk of VF than placebo **(B)**.

Supplementary Figure 2 Forest plot comparing the ratio of patients who subsequently required defibrillation counter shocks (DCSs) after the release of aortic cross-clamp (ACC) during coronary artery bypass surgery. CI, confidence intervals. The rate did not differ significantly between amiodarone and lidocaine groups **(A)**; the percentage of patients requiring DCSs for VF did not differ significantly between patients receiving amiodarone and placebo **(B)**.

## Abbreviations

ACC: aortic cross-clamp
AF: atrial fibrillation
AV: atrioventricular
CABG: coronary artery bypass surgery
CHD: coronary heart disease
CI: confidence intervals
CPB: cardiopulmonary bypass
CPR: cardiopulmonary resuscitation
CVD: cardiovascular disease
DCSs: defibrillation counter shocks
ED: endothelial dysfunction
IHCA: in-hospital sudden cardiac arrest
IRI: ischemia-reperfusion injury
ITT: intention-to-treat
IV: intravenous injection
LVEF: left ventricular ejection fraction
NO: nitric oxide
PRISMA: preferred reporting items for systematic reviews and Meta-analyses
RCT: randomized controlled trial
RVF: reperfusion ventricular fibrillation
RR: risk ratio
TLR-9: Toll-like receptor 9
VF: ventricular fibrillation
VHD: valvular heart disease
VT: ventricular tachycardia
VW: Vaughan-Williams.

## References

[1] Papakonstantinou NA Baikoussis NG Dedeilias P Argiriou M Charitos C. Cardiac surgery or interventional cardiology? Why not both? Let's go hybrid *J Cardiol*. (2017) 1:46–56. 10.1016/j.jjcc.2016.09.007 27727088

[2] Baumann Kreuziger L Karkouti K Tweddell J Massicotte MP. Antithrombotic therapy management of adult and pediatric cardiac surgery patients. J Thromb Haemost. (2018) 11:2133–46. 10.1111/jth.14276 30153372

[3] Geissler H Schlensak C Südkamp M Beyersdorf F. Heart valve surgery today: indications, operative technique, and selected aspects of postoperative care in acquired valvular heart disease. Dtsch Arztebl Int. (2009) 13:224–33; quiz 34. 10.3238/arztebl.2009.0224 19471589 PMC2683397

[4] Tamura T Yatabe T Yokoyama M. Prediction of ventricular fibrillation after release of aortic cross-clamping in cardiovascular surgery patients: a single center retrospective observation study. Anesthesiol Case Rep. (2020) 1:1–6.

[5] Yilmaz M Aydin U Arslan ZI Balci C Kocogullari CU Ata Y . The effect of lidocaine and amiodarone on prevention of ventricular fibrillation in patients undergoing coronary artery bypass grafting. Heart Surg Forum. (2014) 5:E245–9. 10.1532/HSF98.2014402 25367235

[6] Zheng Y Gu Q Chen HW Peng HM Jia DY Zhou Y . Efficacy of amiodarone and lidocaine for preventing ventricular fibrillation after aortic cross-clamp release in open heart surgery: a meta-analysis of randomized controlled trials. J Zhejiang Univ Sci B. (2017) 12:1113–22. 10.1631/jzus.B1700229 29204991 PMC5742294

[7] Mita N Kagaya S Miyoshi S Kuroda M. Prophylactic effect of amiodarone infusion on reperfusion ventricular fibrillation after release of aortic cross-clamp in patients with left ventricular hypertrophy undergoing aortic valve replacement: a randomized controlled trial. J Cardiothorac Vasc Anesth. (2019) 5:1205–13. 10.1053/j.jvca.2018.10.005 30416026

[8] Adameova A Shah A K Dhalla N S. Role of oxidative stress in the genesis of ventricular arrhythmias. Int J Mol Sci. (2020) 12:4200. 10.3390/ijms21124200 32545595 PMC7349053

[9] Samantaray A Chandra A Panigrahi S. Amiodarone for the prevention of reperfusion ventricular fibrillation. J Cardiothorac Vasc Anesth. (2010) 2:239–43. 10.1053/j.jvca.2009.07.007 19800815

[10] Mauermann WJ Pulido JN Barbara DW Abel MD Li Z Meade LA . Amiodarone versus lidocaine and placebo for the prevention of ventricular fibrillation after aortic crossclamping: a randomized, double-blind, placebo-controlled trial. J Thorac Cardiovasc Surg. (2012) 5:1229–34. 10.1016/j.jtcvs.2012.06.039 22770549

[11] Ayoub CM Sfeir PM Bou-Khalil P Azar M Haddadin AS Harfouch D . Prophylactic amiodarone versus lidocaine for prevention of reperfusion ventricular fibrillation after release of aortic cross-clamp. Eur J Anaesthesiol. (2009) 12:1056–60. 10.1097/EJA.0b013e32832f0dfb 19809326

[12] Goyal A Chhabra L Sciammarella JC Cooper JS. Defibrillation. StatPearls. Treasure Island (FL): StatPearls Publishing Copyright © 2020, StatPearls Publishing LLC (2020).

[13] Hippeläinen MJ Tuppurainen TT Huttunen KT. Reperfusion ventricular fibrillation and electric countershocks during coronary artery bypass operations—association with postoperative CK-MB release. Scand J Thorac Cardiovasc Surg. (1994) 2:73–8. 10.3109/14017439409100166 7863289

[14] Kar SK Dasgupta CS Goswami A. Effect of prophylactic amiodarone in patients with rheumatic valve disease undergoing valve replacement surgery. Ann Card Anaesth. (2011) 3:176–82. 10.4103/0971-9784.83986 21860188

[15] Alizadeh-Ghavidel A Nabavi S Haghjoo M Toutonchi Z Mirmesdagh Y Javadikasgari H. Amiodarone versus lidocaine for the prevention of reperfusion ventricular fibrillation: A randomized clinical trial. ARYA Atheroscler. (2013) 6:343–9. 24575137 PMC3933055

[16] Page MJ McKenzie JE Bossuyt PM Boutron I Hoffmann TC Mulrow CD . The PRISMA statement: an updated guideline for reporting systematic reviews. BMJ. (2021) 372:n71. 10.1136/bmj.n71 33782057 PMC8005924

[17] Furlan AD Malmivaara A Chou R Maher CG Deyo RA Schoene M . 2015 Updated Method Guideline for Systematic Reviews in the Cochrane Back and Neck Group. Spine (Phila Pa 1976). (2015) 21:1660–73. 10.1097/BRS.0000000000001061 26208232

[18] Fu DL Lu L Zhu W Li JH Li HQ Liu AJ . Xiaoxuming decoction for acute ischemic stroke: a systematic review and meta-analysis. J Ethnopharmacol. (2013) 1:1–13. 10.1016/j.jep.2013.04.002 23583540

[19] Komori S Li B Matsumura K Takusagawa M Sano S Kohno I . Antiarrhythmic effect of magnesium sulfate against occlusion-induced arrhythmias and reperfusion-induced arrhythmias in anesthetized rats. Mol Cell Biochem. (1999) 1–2:201–8. 10.1023/A:1006938010925 10544968

[20] Nayeem Ul. Hassan, Dar AM, Wani ML, Rather HA, Ganie FA. A comparative study on the effect of amiodarone and metaprolol for prevention of arrythmias after open heart surgery. Int Cardiovasc Res J. (2013) 1:1–4. PMC3987421 24757610

[21] Wyman MG Wyman RM Cannom DS Criley JM. Prevention of primary ventricular fibrillation in acute myocardial infarction with prophylactic lidocaine. Am J Cardiol. (2004) 5:545–51. 10.1016/j.amjcard.2004.05.014 15342281

[22] Rea RS Kane-Gill SL Rudis MI Seybert AL Oyen LJ Ou NN . Comparing intravenous amiodarone or lidocaine, or both, outcomes for inpatients with pulseless ventricular arrhythmias. Crit Care Med. (2006) 6:1617–23. 10.1097/01.CCM.0000217965.30554.D8 16614583

[23] Wang CH Chang WT Huang CH Tsai MS Yu PH Wu YW . Outcomes associated with amiodarone and lidocaine for the treatment of adult in-hospital cardiac arrest with shock-refractory pulseless ventricular tachyarrhythmia. J Formos Med Assoc. (2020) 1 Pt 2:327–34. 10.1016/j.jfma.2019.05.023 31255419

[24] Elnakera A Alawady TA. Continuous infusion of magnesium–lidocaine mixture for prevention of ventricular arrhythmias during on-pump coronary artery bypass grafting surgery. Egypt J Anaesthesia. (2013) 4:419–25. 10.1016/j.egja.2013.05.002

[25] Petrovic T Adnet F Lapandry C. Successful resuscitation of ventricular fibrillation after low-dose amiodarone. Ann Emerg Med. (1998) 4:518–9. 10.1016/S0196-0644(98)70191-X 9774944

[26] Zoerner F Semenas E. Resuscitation with amiodarone increases survival after hemorrhage and ventricular fibrillation in pigs. J Trauma Acute Care Surg. (2014) 6:1402–8. 10.1097/TA.0000000000000243 24854308

[27] Lee DK Kim YJ Kim G Lee CA Moon HJ Oh J . Impact of early intravenous amiodarone administration on neurological outcome in refractory ventricular fibrillation: retrospective analysis of prospectively collected prehospital data. Scand J Trauma Resusc Emerg Med. (2019) 1:109. 10.1186/s13049-019-0688-1 31823800 PMC6902320

[28] Wu L Jin Q Zhang N Pang Y Ren S Zhou J . The effects of acute amiodarone on short- and long-duration ventricular defibrillation threshold in canines. J Cardiovasc Pharmacol. (2011) 4:432–8. 10.1097/FJC.0b013e318228a50c 21709582

[29] Chevalier P Timour Q Morel E Bui-Xuan B. Chronic oral amiodarone but not dronedarone therapy increases ventricular defibrillation threshold during acute myocardial ischemia in a closed-chest animal model. J Cardiovasc Pharmacol. (2012) 6:523–8. 10.1097/FJC.0b013e31824d89fe 22330675

[30] McLeod SL Iansavichene A Cheskes S. Remote Ischemic Perconditioning to Reduce Reperfusion Injury During Acute ST-Segment-Elevation Myocardial Infarction: A Systematic Review and Meta-Analysis. J Am Heart Assoc. (2017) 5. 10.1161/JAHA.117.005522 28515120 PMC5524098

[31] Vinten-Johansen J Yellon DM Opie LH. Postconditioning: a simple, clinically applicable procedure to improve revascularization in acute myocardial infarction. Circulation. (2005) 14:2085–8. 10.1161/CIRCULATIONAHA.105.569798 16203924

[32] Ma J Qiao Z Xu B. Effects of ischemic preconditioning on myocardium Caspase-3, SOCS-1, SOCS-3, TNF-α and IL-6 mRNA expression levels in myocardium IR rats. Mol Biol Rep. (2013) 10:5741–8. 10.1007/s11033-013-2677-1 24091940

[33] Naase H Harling L Kidher E Sepehripour A Nguyen B Kapelouzou A . Toll-like receptor 9 and the inflammatory response to surgical trauma and cardiopulmonary bypass. J Cardiothorac Surg. (2020) 1:137. 10.1186/s13019-020-01179-y 32527277 PMC7291696

[34] Iwamoto T Watanabe Y Kita S Blaustein MP. Na+/Ca2+ exchange inhibitors: a new class of calcium regulators. Cardiovasc Hematol Disord Drug Targets. (2007) 3:188–98. 10.2174/187152907781745288 17896959

