# Supplementary figures and images for: Effectiveness of Amiodarone in Preventing the Occurrence of Reperfusion Ventricular Fibrillation After the Release of Aortic Cross-Clamp in Open-Heart Surgery Patients: A Meta-Analysis
Source: Front Cardiovasc Med. 2022 Feb 4;9:821938. doi: 10.3389/fcvm.2022.821938 (PMC8854653; doi:10.3389/fcvm.2022.821938)

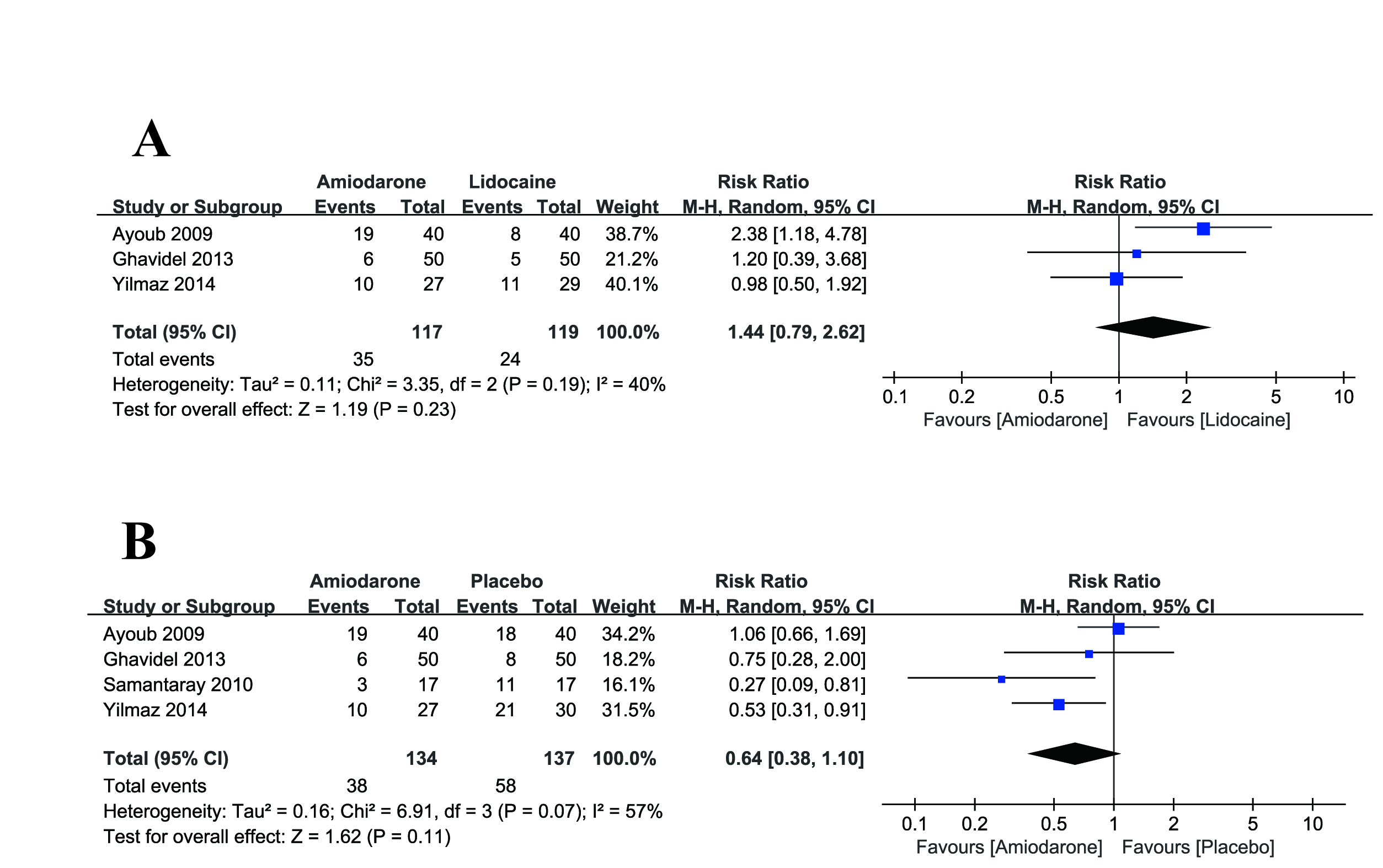

Supplement: Supplementary Figure 1 — Forest plot comparing the incidence of ventricular fibrillation (VF) after the release of aortic cross-clamp (ACC) in patients undergoing coronary artery bypass surgery (CABG) who were treated with amiodarone, lidocaine or placebo. CI, confidence intervals. The rate of VF after release of ACC did not differ significantly between patients undergoing open heart surgery who were treated with amiodarone or lidocaine (A); amiodarone was not associated with a lower risk of VF than placebo (B). [file Figure_1.TIF]

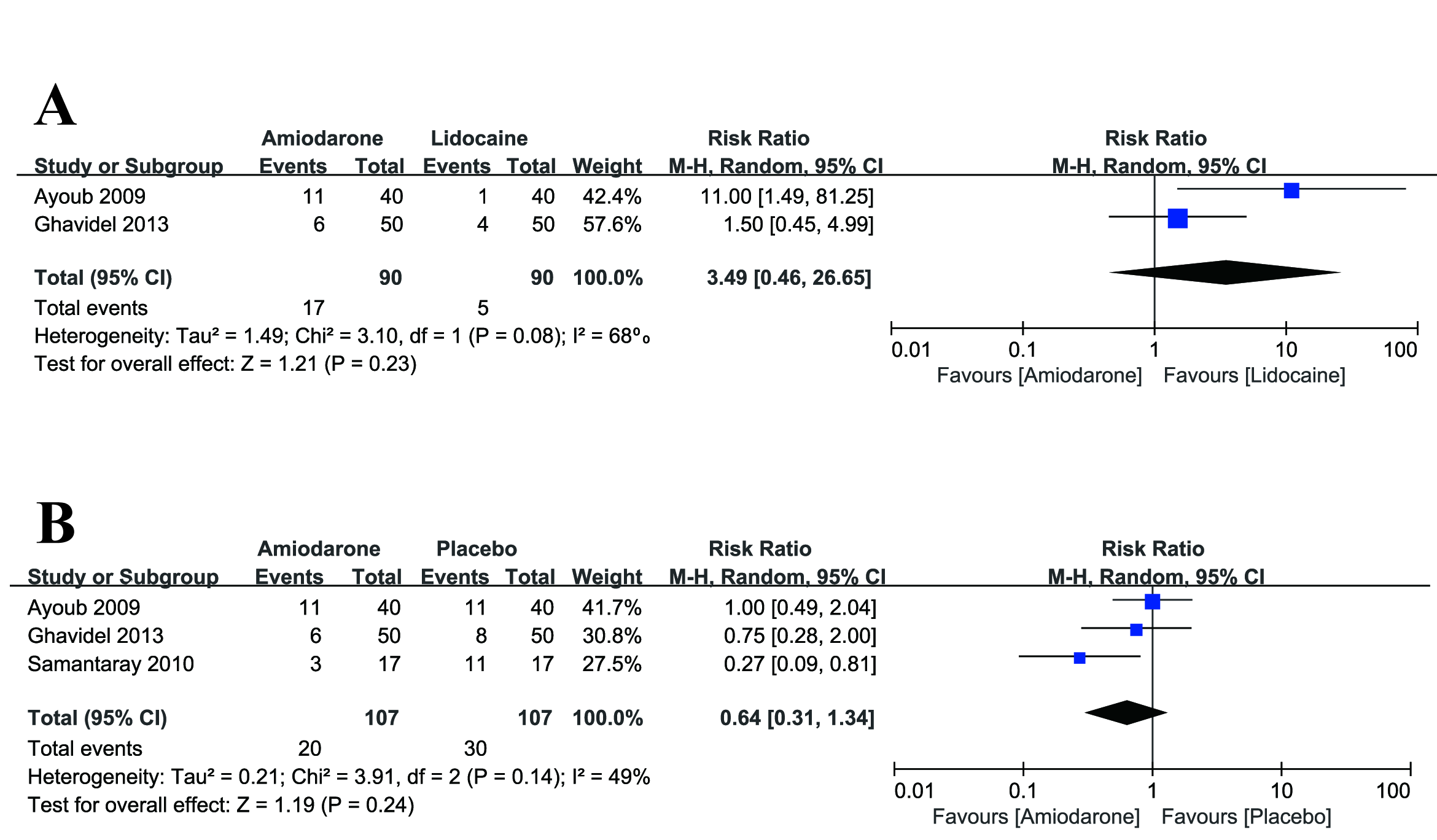

Supplement: Supplementary Figure 2 — Forest plot comparing the ratio of patients who subsequently required defibrillation counter shocks (DCSs) after the release of aortic cross-clamp (ACC) during coronary artery bypass surgery. CI, confidence intervals. The rate did not differ significantly between amiodarone and lidocaine groups (A); the percentage of patients requiring DCSs for VF did not differ significantly between patients receiving amiodarone and placebo (B). [file Figure_2.TIF]
